# Tomographic findings in bronchial atresia

**DOI:** 10.1590/0100-3984.2019.0136

**Published:** 2021

**Authors:** Elazir Barbosa Mota Di Puglia, Rosana Souza Rodrigues, Pedro Augusto Daltro, Arthur Soares Souza Jr., Marilene Monteiro Paschoal, Ester Moraes Labrunie, Klaus Loureiro Irion, Bruno Hochhegger, Gláucia Zanetti, Edson Marchiori

**Affiliations:** 1 Universidade Federal do Rio de Janeiro (UFRJ), Rio de Janeiro, RJ, Brazil.; 2 Clínica de Diagnóstico por Imagem (CDPI), Rio de Janeiro, RJ, Brazil.; 3 Instituto D’Or de Pesquisa e Ensino (IDOR), Rio de Janeiro, RJ, Brazil.; 4 Faculdade de Medicina de São José do Rio Preto (Famerp), São José do Rio Preto, SP, Brazil.; 5 The Liverpool Heart and Chest Hospital, NHS Trust, Liverpool, UK.; 6 Universidade Federal de Ciências da Saúde de Porto Alegre (UFCSPA), Porto Alegre, RS, Brazil.

**Keywords:** Bronchi/abnormalities, Congenital abnormalities, Tomography, X-ray computed, Brônquios/anormalidades, Anomalias congênitas, Tomografia computadorizada

## Abstract

**Objective:**

To evaluate computed tomography (CT) findings in 23 patients with bronchial atresia.

**Materials and Methods:**

The CT images were reviewed by two radiologists who reached decisions by consensus. We included only patients who presented with abnormalities on CT and in whom the diagnosis had been confirmed by pathological examination of the surgical specimen (if the lesion was resected). The CT scans were assessed in order to identify the main findings and to map the distribution of the lesions (i.e., to determine whether the pulmonary involvement was unilateral or bilateral).

**Results:**

The main CT finding was the combination of bronchocele and hyperinflation of the distal lung. That combination was observed in all of the patients. The lesions were unilateral in all 23 cases, being seen predominantly in the left upper lobe, followed by the right lower lobe, right upper lobe, middle lobe, and left lower lobe.

**Conclusion:**

The diagnosis of bronchial atresia can be reliably made on the basis of a finding of bronchocele accompanied by hyperinflation of the adjacent lung parenchyma.

## Abstract (pt)

**Objetivo:**

Analisar os achados na tomografia computadorizada (TC) de tórax de 23 pacientes com atresia brônquica.

**Materiais e Métodos:**

As imagens de TC foram avaliadas por dois observadores e os casos discordantes foram resolvidos por consenso. Os critérios de inclusão foram a presença de anormalidades na TC compatíveis com atresia brônquica e/ou diagnóstico confirmado por exame anatomopatológico das peças cirúrgicas para os pacientes submetidos a ressecção cirúrgica. As TCs foram avaliadas quanto aos principais achados de imagem, à distribuição das lesões, ao envolvimento pulmonar unilateral ou bilateral.

**Resultados:**

Os principais achados na TC foram a presença de broncocele, hiperinsuflação do parênquima pulmonar ou ambos. A combinação desses achados foi encontrada em todos os pacientes. Em relação à distribuição, o envolvimento foi unilateral nos 23 casos. Quando se consideraram os lobos mais acometidos, o lobo superior esquerdo foi o mais acometido, seguido do lobo inferior direito, lobo superior direito, lobo médio e lobo inferior esquerdo.

**Conclusão:**

O diagnóstico de atresia brônquica pode ser feito em presença de broncocele associada com hiperinsuflação do parênquima pulmonar adjacente.

## INTRODUCTION

Bronchial atresia is a rare congenital disease, characterized by the obliteration of the proximal lumen of a lobar, segmental, or subsegmental bronchus, with preservation of distal structures^(1-3)^. It most commonly affects the apicoposterior segment of the left upper lobe, followed by the segmental bronchi of the right upper and middle lobes. The involvement of the lower lobes is uncommon^(4-8)^. In most cases, the disease is benign and asymptomatic, being discovered only as an incidental finding in adulthood^(3,6,8)^. In rare cases, children and adolescents can be symptomatic and present with recurrent episodes of pneumonia^(7)^. 

Computed tomography (CT) is the examination of choice to confirm the presence of an abnormality, showing mucoid impaction and hyperinflation of the distal lung parenchyma^(6,9,10)^.

The treatment for bronchial atresia remains controversial; however, because most patients are asymptomatic, observation (watchful waiting) is the preferred option^(6)^.

In the present study, we analyzed the chest CT scans of patients with bronchial atresia, to assess the most common CT findings, as well as to map the distribution of the lesions and evaluate their morphological characteristics. In addition, we studied some epidemiological aspects, such as distribution by gender and age.

## MATERIALS AND METHODS

This was a retrospective study of the chest CT scans of 23 patients with bronchial atresia. The examinations were collected randomly via contact with radiologists at eight different institutions in four Brazilian states (Rio de Janeiro, São Paulo, Minas Gerais, and Rio Grande do Sul) and in Argentina. The diagnoses were confirmed on the basis of a combination of clinical and radiological data, as well as, in a few cases, histopathological data. The inclusion criteria were either the presence of typical abnormalities in the CT, a confirmed diagnosis by pathological examination of surgical specimens (if the lesion was resected), or both.

Because so many different institutions were involved, the chest CT scans were acquired in different scanners, although volumetric acquisition was performed in all cases. The CT scans were acquired with fine axial slices, 1-2 mm in thickness (from the lung apices to the lung bases), with the patient in the supine position, during inspiration, with a high spatial resolution algorithm to reconstruct the images. In some cases, iodinated contrast medium was administered intravenously. The images were obtained and reconstructed in a 512 × 512 matrix, with lung window settings (with a width of 1000-1500 HU and a level between -650 HU and -750 HU), and mediastinal window settings (with a width of 350-400 HU and a level between 40 HU and 60 HU).

The CT images were assessed for the following aspects: mucocele; hyperinflation of the parenchyma; atelectasis; bronchial wall thickening; nodules in the airspace; consolidations; and cysts. A mucocele was characterized as an area of bronchial dilation with retention of secretion, presenting as either a tubular or branching image that is similar to the finger of a glove (the finger-in-glove sign). Lung hyperinflation was defined as a reduction in the attenuation of the lung parenchyma, accompanied by a reduction of vascular structures (hypovolemia), with or without increased lung volume. Laminar atelectasis was defined as a focus of subsegmental atelectasis with a linear or discoid configuration, almost always extending to the pleura. Nodules in the airspace were defined as those that were smaller than 1 cm, with ill-defined contours, and tended to converge. Consolidation was defined as an increase in the attenuation of the lung parenchyma that impedes the visualization of the blood vessels and the external contours of the bronchial walls. Cysts were defined as any rounded, well circumscribed space that is surrounded by an epithelialized or fibrous wall of variable thickness. The defining criteria of those findings are those reported in the “Fleischner Society: glossary of terms for thoracic imaging”^(11)^, and the terms used here are those suggested in the terminology consensus statement of the Department of Imaging of the Brazilian Thoracic Association^(12)^.

The morphology, content, and distribution of the mucoceles were also described. As for morphology, when a mucocele presented only one lobulation it was defined as oval or round in shape, and when it presented two or more lobulations, it was defined as branching. As for content, it was defined as mucoid when filled with a material of fluid density and as aerial when completely filled with air. All of the lesions were mapped for their distribution in the lung parenchyma. Regarding distribution, they were classified as either unilateral or bilateral, and regarding their lobar distribution as right upper, left upper, middle, right lower, and left lower. The examinations were interpreted by two radiologists, and any disagreements were resolved by consensus.

## RESULTS

### Clinical and epidemiological aspects

We evaluated the chest CT scans of 23 patients with bronchial atresia, of whom 12 (52%) were male and 11 (48%) were female. Ages ranged from 2 months to 69 years (mean, 34.7 years), and eight of the patients were under 20 years of age. Among the female patients, ages ranged from 2 months to 67 years (mean, 38.9 years), whereas they ranged from 4 to 69 years (mean, 29.8 years) among the male patients. Of the 23 patients, 17 (74%) were asymptomatic and were undergoing the examination for other reasons. Cough and recurrent pneumonia were reported by three patients (13%) each.

### Patterns on CT

**Figure 1:**
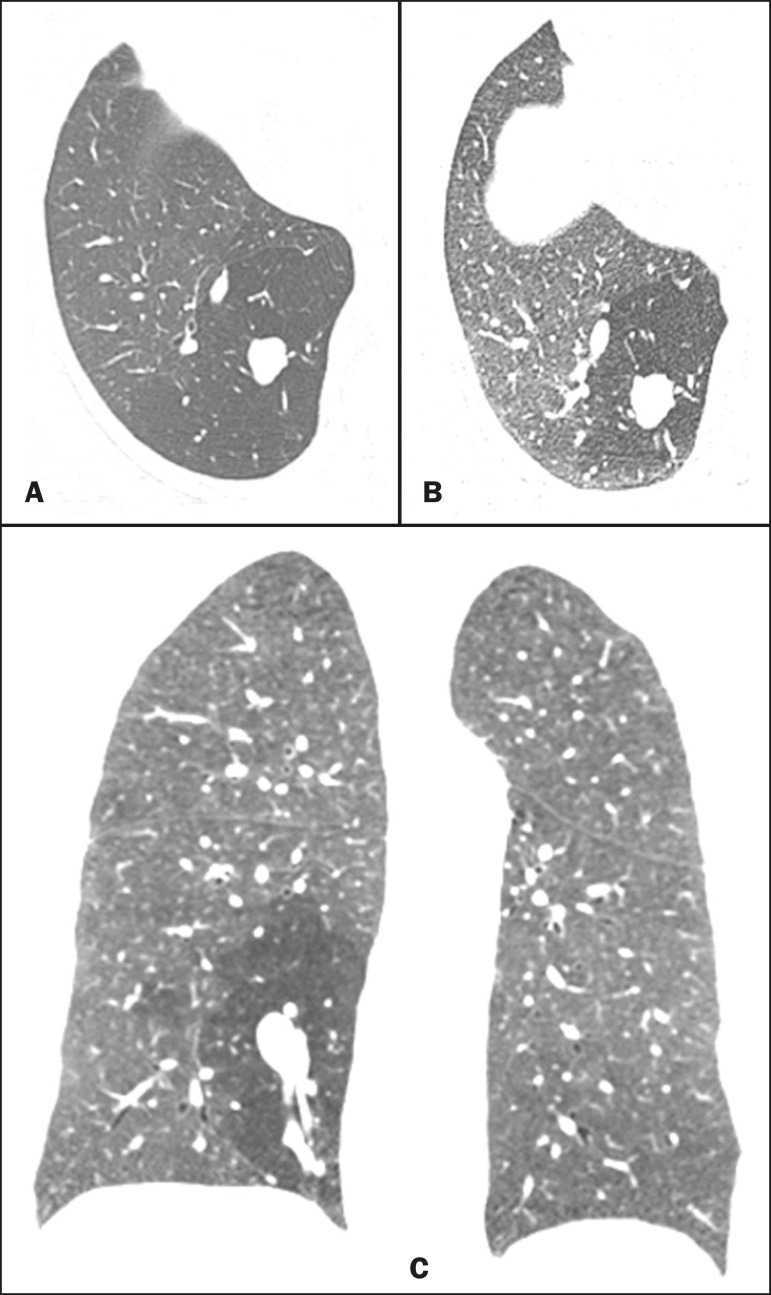
Female, 28-year-old, asymptomatic. Axial chest CT scan with lung window settings, at the level of the pulmonary bases during inspiration (**A**) and expiration (**B**), together with a coronal slice acquired during expiration (**C**), showing a lobulated nodule (mucocele), in the posterior basal segment of the right lower lobe, with an area of hyperinflation around the lesion (air trapping), best visualized in the expiratory sequences (**B,C**).

**Figure 2:**
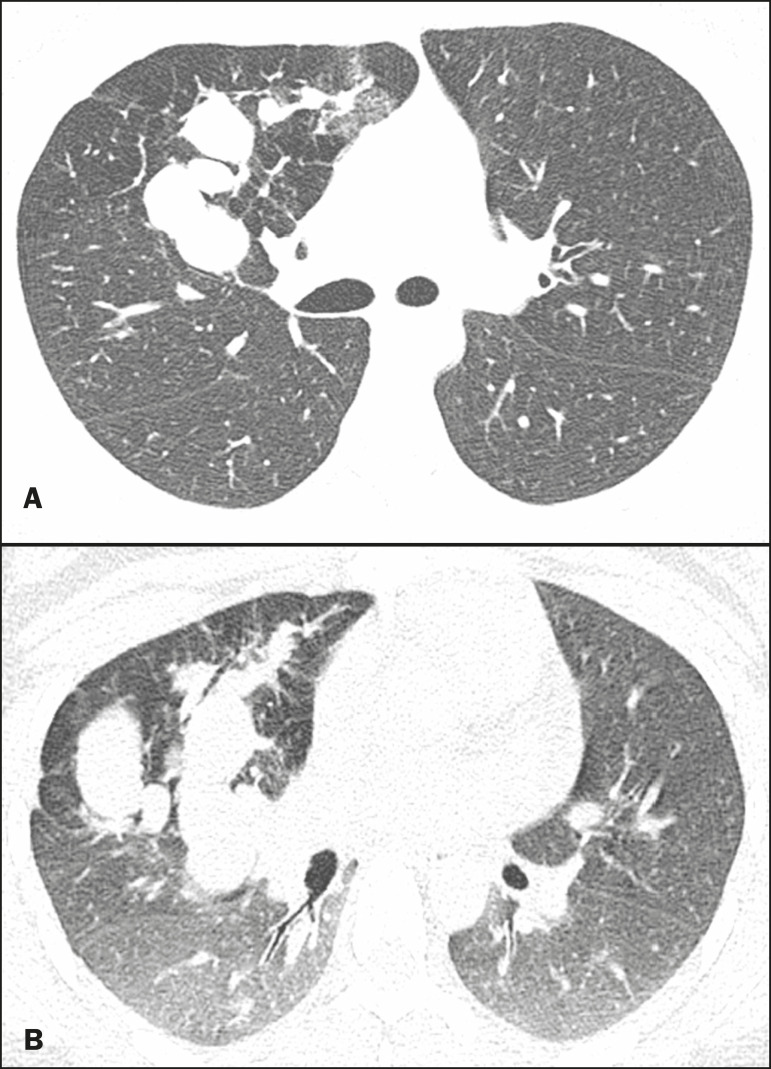
Male, 27-year-old, presenting with cough symptoms. Axial chest CT scan (**A**), showing branching opacities in the right upper lobe, with adjacent lung hyperinflation, best visualized in an expiratory sequence (**B**).

**Figure 3:**
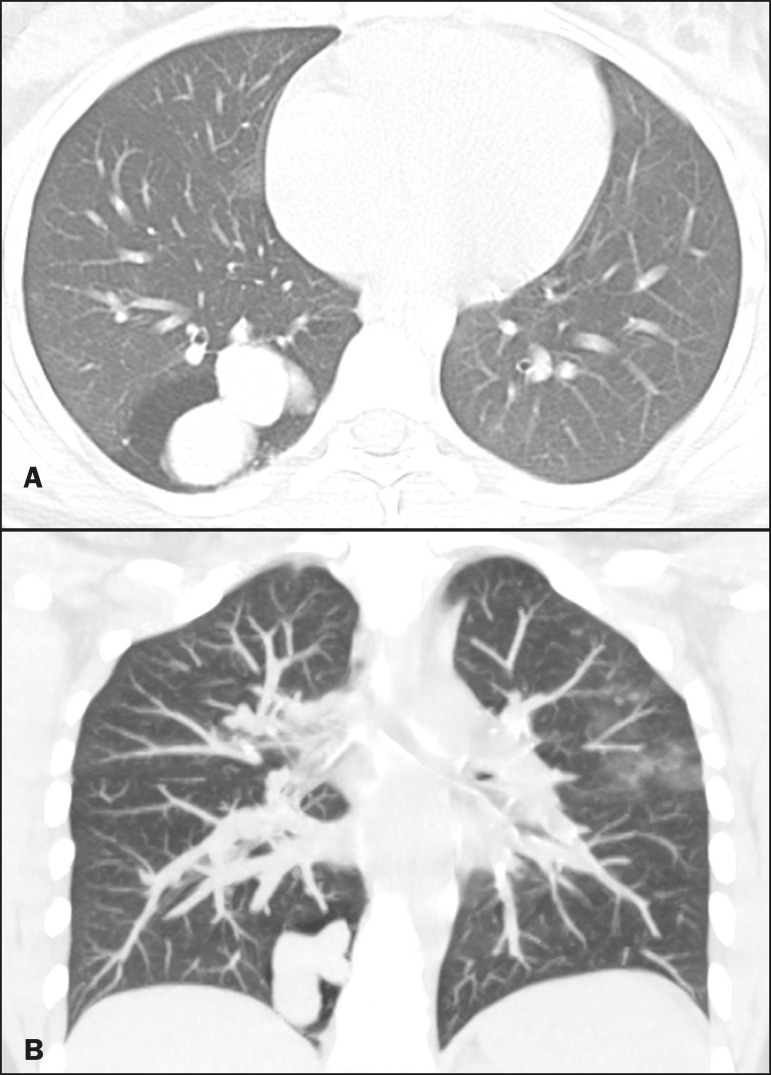
Male, 30-year-old, asymptomatic. Axial CT scan (**A**), showing nodular opacities (mucoceles) in the right lower lobe, with hyperinflation of the adjacent lung parenchyma. Coronal reconstruction (**B**), better showing the branching of the mucocele.

**Figure 4:**
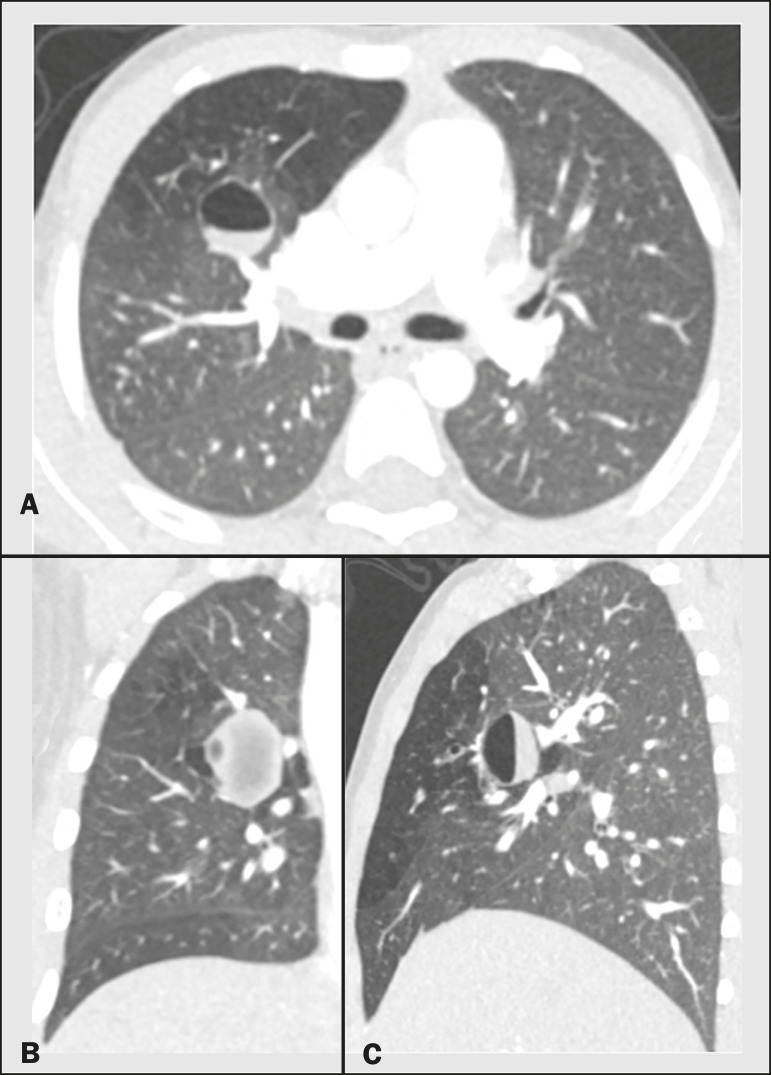
Boy, 7-year-old, asymptomatic. Axial, coronal, and sagittal slices (**A**, **B**, and **C**, respectively), showing a nodule with a fluid-fluid level (mucocele) in the right upper lobe, together with air trapping in the adjacent lung parenchyma.

The tomographic findings observed in the sample were, in decreasing order of frequency: mucoceles, in all 23 patients (100%); hyperinflation of the adjacent lung parenchyma, also in all 23 (100%); subsegmental atelectasis, in six (26%); bronchial wall thickening, in four (17%); nodules in the airspace, in two (8%); consolidations, in one (4%); and small cysts, in one (4%) (Figures 1-4). Nodular airspace opacities were seen in two patients who had a history of recurrent pneumonia, one of them undergoing the examination to evaluate the improvement of their respiratory condition. Consolidation was observed in one patient who had undergone treatment for community-acquired pneumonia, and that patient subsequently developed a cough, later presenting other recurrent respiratory conditions. In another patient, there were small lung cysts adjacent to the area of bronchial atresia. The pathological analysis of that case confirmed its association with congenital pulmonary airway malformation (CPAM), characterizing the lesion as hybrid.

### Aspect of the mucoceles

The mucoceles varied in terms of shape and air content. The mucoceles were branching in 13 cases (57%) and oval or in the remaining 10 (43%). In 21 patients (91%), the mucocele was completely filled with mucus, whereas it was completely filled with air in the 2 remaining patients (9%).

### Distribution of the CT findings

The involvement was unilateral in all 23 cases (100%), being right-sided in 14 (61%) and left-sided in 9 (39%). As shown in Figure 1, the most commonly affected lobe was the left upper lobe, in 8 cases (35%), followed by the right lower lobe, in 6 (26%), right upper lobe, in 4 (17%), middle lobe, in 4 (17%), and left lower lobe, in 1 (5%).

## DISCUSSION

### Clinical and epidemiological findings

Bronchial atresia is a rare congenital pulmonary disease, characterized by the obliteration of the proximal lumen of a lobar, segmental, or subsegmental bronchus, with preservation of distal structures. It generally follows a benign, asymptomatic course^(4,6,13)^. Bronchial atresia is slightly more prevalent in males, and its reported incidence varies in the literature^(6,13,14)^. In the present study, the incidence was the same for both genders. The mean age of the patients in our sample-34.7 years-is in agreement with data in the literature showing that the diagnosis is typically made only in the second or third decade of life. That probably occurs because most patients are asymptomatic and are diagnosed when undergoing examinations for other reasons^(14,15)^.

As previously mentioned, most patients with bronchial atresia are asymptomatic, and the findings on physical examination are minimal^(1,2,10)^. Although rare, respiratory symptoms such as infection, cough, and tachypnea can occur, and those symptoms are most commonly seen in neonates and preschool children^(7)^. Patients over seven years of age are typically asymptomatic. Of the 23 patients in our sample, 17 (74%) were asymptomatic and had undergone the examination for other reasons. Respiratory symptoms were present in six patients (26%): recurrent cough in three (13%) and pneumonia in three (13%). In a study involving 12 patients with bronchial atresia, Wang et al.^(3)^ reported that 7 (58%) were asymptomatic. In the remaining 5 patients (42%), the symptoms included fever, cough, hemoptysis, and dyspnea. Matsushima et al.^(9)^ analyzed the CT scans of nine patients with bronchial atresia and found that six (66%) were asymptomatic, two (22%) had a cough, and one (12%) had dyspnea. Those data are in agreement with our findings.

### CT findings

Evaluation of thoracic diseases through CT has been the motive for a series of recent publications in the radiology literature of Brazil^(16-23)^. Chest CT is also the method of choice for diagnosing bronchial atresia, because it is the most sensitive in showing the findings typical of the disease, such as lung hyperinflation. A CT scan is also useful to exclude the presence of hilar masses and show the bronchoceles that are not impregnated by the contrast medium^(6,9,10)^. On chest X-rays, it is often difficult to identify a mucocele, especially if the bronchial obstruction is subsegmental or accompanied by an infectious process^(9)^.

Mucocele, distal hyperinflation, and hypovascularization (hypovolemia) seen on CT are characteristics of the disease^(3,5,9,10,24,25)^. Therefore, most authors agree that the presence of these CT findings supports the diagnosis of bronchial atresia^(9)^. Concurrent infections can be accompanied by nodules in the airspace and consolidations^(9)^. In the present study, the most common findings were hyperinflation of the lung parenchyma and mucocele, findings similar to those in the literature.

Other findings observed in our patients were atelectasis, found in six patients (26%), bronchial wall thickening, in four patients (17%), and nodules in the airspace, in two patients (8%). Consolidations were observed in only one patient (4%) and were probably related to a secondary infection. Small pulmonary cysts, adjacent to the area of bronchial atresia, were observed in one other patient (4%). The pathological analysis confirmed an association with CPAM, characterizing the lesion as hybrid. The strong association between CPAM and bronchial atresia has been well established in the literature, the combination of the two occurring in approximately 70% of cases^(26,27)^. When there is obstruction of the bronchus during intrauterine life, the adjacent lung parenchyma becomes dysplastic, and there is an association between the two conditions. In this context, the appearance of cysts should not be viewed as a separate condition but as a common consequence of the obstruction of the airways during early pulmonary development^(27,28)^. Kunisaki et al.^(4)^ evaluated 25 patients and found that bronchial atresia was accompanied by other congenital pulmonary malformations in 77% of those patients, the combination of CPAM and congenital pulmonary hyperinflation being predominant. Newman^(28)^ and Bush^(29)^ both recently proposed that all congenital lesions be grouped together and named more generically as congenital pulmonary malformations, because of the important associations among them. 

When we analyzed the morphology of the mucoceles, the branching pattern was predominant, being observed in 13 cases (57%), whereas the mucoceles were oval or rounded in the remaining 10 cases (43%). In 21 patients (91%) the mucoceles were completely filled with mucus, whereas they were completely filled with air in 2 (9%). Matsushima et al.^(9)^ also analyzed the characteristics of mucoceles and obtained similar data, observing predominance of the branching form and of fluid content.

In the literature, it has been reported that bronchial atresia most often affects the left upper lobe, followed by the right upper, middle, and lower lobes^(6,8)^. In the present study, the left upper lobe was affected in eight cases (35%), followed by the right lower lobe, in six (26%), the right upper lobe, in four (17%), the middle lobe, in four (17%), and the left lower lobe, in one (5%). Matsushima et al.^(9)^ evaluated nine patients with bronchial atresia and found that it affected the upper lobes in all nine, predominantly affecting the left lung. In a case report and review of the literature, involving a collective total of 35 patients with bronchial atresia evaluated with CT, Ouzidane et al.^(30)^ observed involvement of the left upper lobe in 24 patients (68%), the right lower lobe in four (11%), the left lower lobe in three (10%), the right upper lobe in three (10%), and the middle lobe in one (3%). In our sample, the involvement was unilateral in all cases, being right-sided in 14 patients (61%). In the literature, the data on laterality vary. Wang et al.^(3)^ studied 12 patients and also found that the involvement was unilateral in all of them, being right-sided in 8 (66.6%), which is very similar to our results.

Our study has some limitations. First, it was a retrospective study. In addition, because of the long-term aspect of the study, as well as the numerous institutions and collaborators involved, the patients were examined with a variety of CT techniques. Furthermore, in most cases, images were not acquired in the expiratory phase, and that can limit the ability of CT to detect air trapping, although we do not believe that this had any impact on the results. Moreover, clinical and follow-up data were unavailable for some patients, which made it impossible to make the proper clinical correlation.

In our study, the most common CT finding, seen in all of the patients evaluated, was the combination of hyperinflation of the lung parenchyma and mucocele.

## References

[1] Lee EY, Boiselle PM, Cleveland RH (2008). Multidetector CT evaluation of congenital lung anomalies. Radiology.

[2] Lee EY, Dorkin H, Vargas SO (2011). Congenital pulmonary malformations in pediatric patients review and update on etiology, classification, and imaging findings. Radiol Clin N Am.

[3] Wang Y, Dai W, Sun Y (2012). Congenital bronchial atresia diagnosis and treatment. Int J Med Sci.

[4] Kunisaki SM, Fauza DO, Nemes LP (2006). Bronchial atresia the hidden pathology within a spectrum of prenatally diagnosed lung masses. J Pediatr Surg.

[5] Faure MCA, Barreto APA, Pereira CAC (2000). Atresia brônquica congênita relato de dois casos. Contribuição da tomografia computadorizada ao diagnóstico. J Pneumol.

[6] Gipson MG, Cummings KW, Hurth KM (2009). Bronchial atresia. Radiographics.

[7] Morikawa N, Kuroda T, Honna T (2005). Congenital bronchial atresia in infants and children. J Pediatr Surg.

[8] Praticò FE, Corrado M, Della Casa G (2016). Imaging of congenital pulmonary malformations. Acta Biomed.

[9] Matsushima H, Takayanagi N, Satoh M (2002). Congenital bronchial atresia radiologic findings in nine patients. J Comput Assist Tomogr.

[10] Neu AS, Menezes RE, Ilha DO (2003). Aspectos radiológicos da atresia brônquica relato de três casos e revisão da literatura. Radiol Bras.

[11] Hansell DM, Bankier AA, MacMahon H (2008). Fleischner Society glossary of terms for thoracic imaging. Radiology.

[12] Sociedade Brasileira de Pneumologia e Tisiologia (2005). Diretrizes da SBPT Consenso brasileiro sobre terminologia dos descritores de tomografia computadorizada do tórax. J Bras Pneumol.

[13] Meng RL, Jensik RJ, Faber LP (1978). Bronchial atresia. Ann Thorac Surg.

[14] Traibi A, Seguin-Givelet A, Grigoroiu M (2017). Congenital bronchial atresia in adults thoracoscopic resection. J Vis Surg.

[15] Heidinger BH, Occhipinti M, Eisenberg RL (2015). Imaging of large airways disorders. AJR Am J Roentgenol.

[16] Guataqui AEC, Muniz BC, Niemeyer B (2019). Hamman's syndrome accompanied by pneumorrhachis. Radiol Bras.

[17] Avelino EBP, Verza L, Neves T (2019). Lymphocytic interstitial pneumonia and pulmonary amyloidosis in Sjögren's syndrome. Radiol Bras.

[18] Lima CMAO (2020). Information about the new coronavirus disease (COVID-19). Radiol Bras.

[19] Torres PPTS, Rabahi MF, Moreira MAC (2018). Tomographic assessment of thoracic fungal diseases a pattern and signs approach. Radiol Bras.

[20] Geraldino ACC, Marchiori E (2019). Cavitary rheumatoid nodules an unusual pulmonary finding. Radiol Bras.

[21] Tibana TK, Camilo DMR, Nunes TF (2019). Congenital lobar emphysema. Radiol Bras.

[22] Santos RFT, Tibana TK, Adôrno IF (2019). Mounier-Kuhn syndrome an unusual cause of bronchiectasis. Radiol Bras.

[23] Adôrno IF, Santos RFT, Faria BB (2019). Pleuropulmonary blastoma manifesting as spontaneous pneumothorax an unusual presentation. Radiol Bras.

[24] Mahajan AK, Rahimi R, Vanderlaan P (2017). Unique approach to diagnosing and treating congenital bronchial atresia (CBA) a case series. J Pulm Respir Med.

[25] Marchiori E, Hochhegger B, Zanetti G (2019). Hyperinflation surrounding a solitary nodule. J Bras Pneumol.

[26] Daltro P, Werner H, Gasparetto TD (2010). Congenital chest malformations a multimodality approach with emphasis on fetal MR imaging. Radiographics.

[27] Rappaport DC, Herman SJ, Weisbrod GL (1994). Congenital bronchopulmonary diseases in adults CT findings. AJR Am J Roentgenol.

[28] Newman B (2006). Congenital bronchopulmonary foregut malformations concepts and controversies. Pediatr Radiol.

[29] Bush A (2001). Congenital lung disease a plea for clear thinking and clear nomenclature. Pediatr Pulmonol.

[30] Ouzidane L, Benjelloun A, el Hajjam M (1999). Segmental bronchial atresia-a case report and a literature review. Eur J Pediatr Surg.

